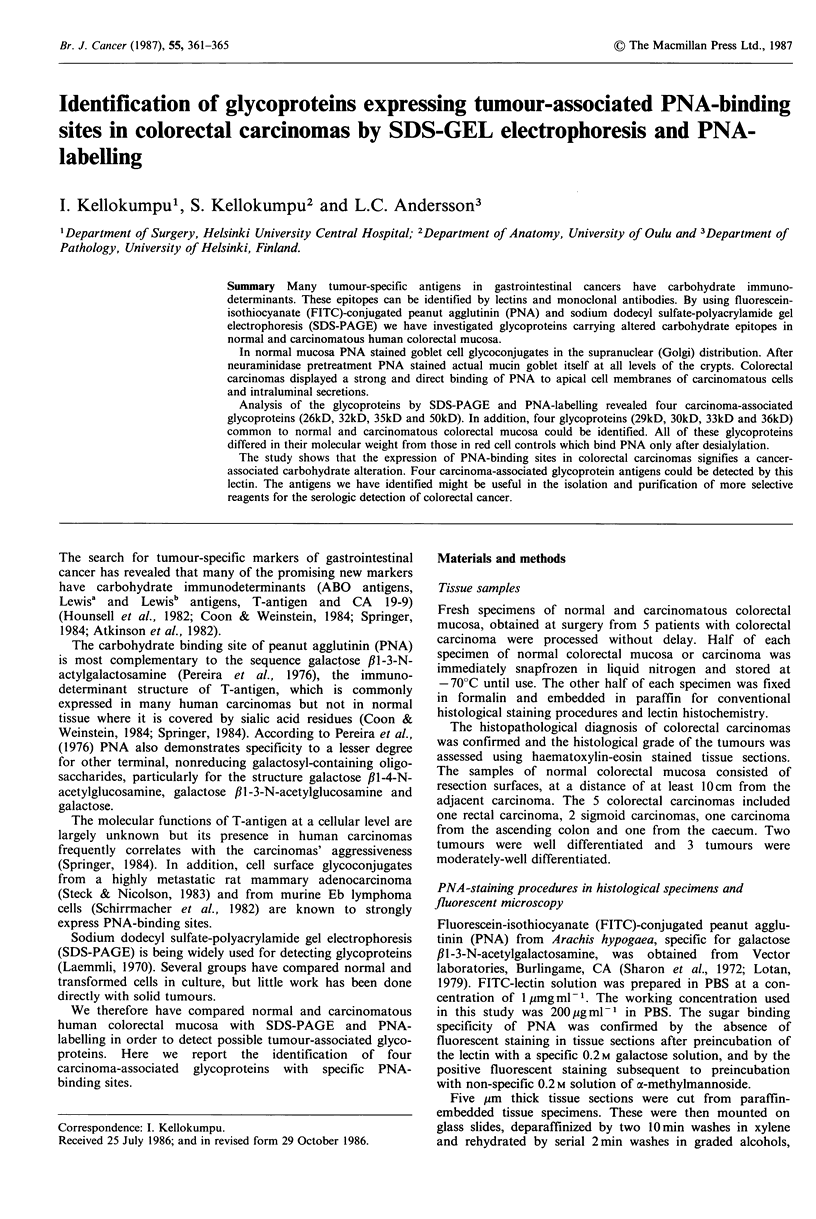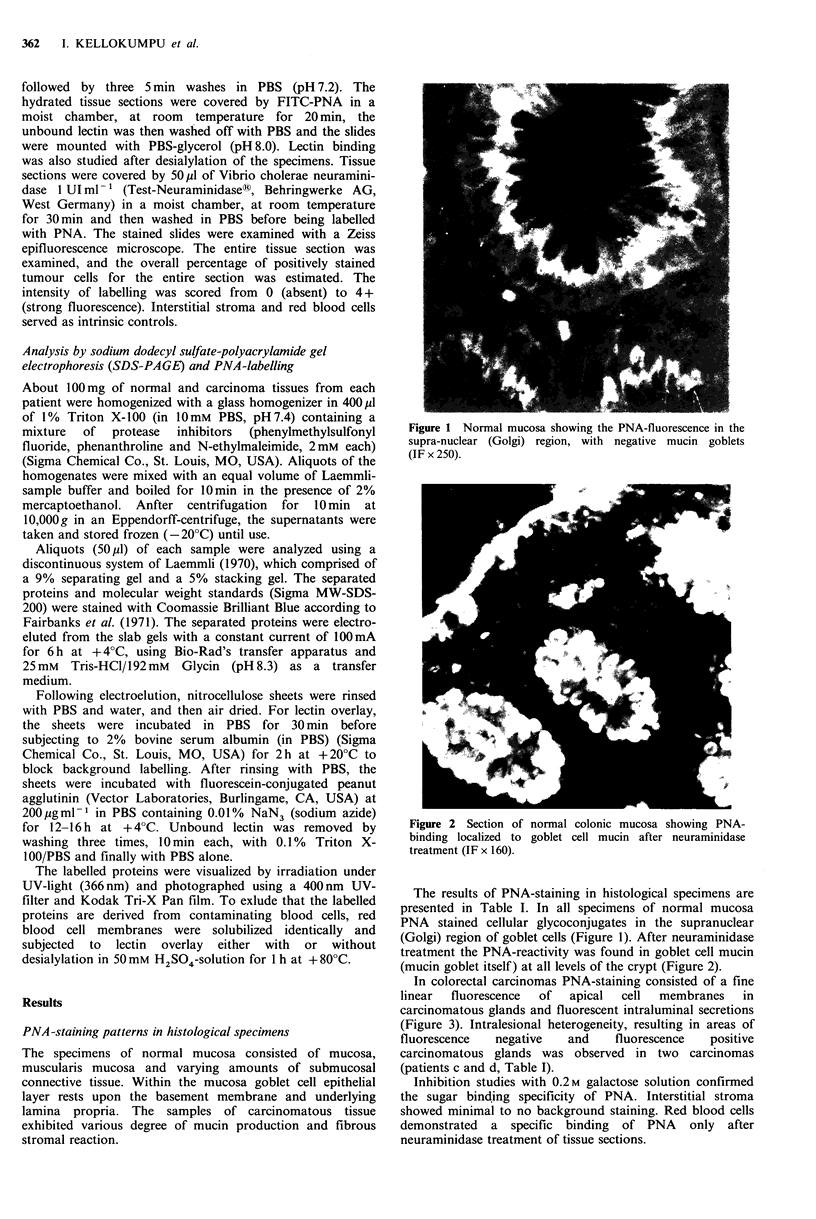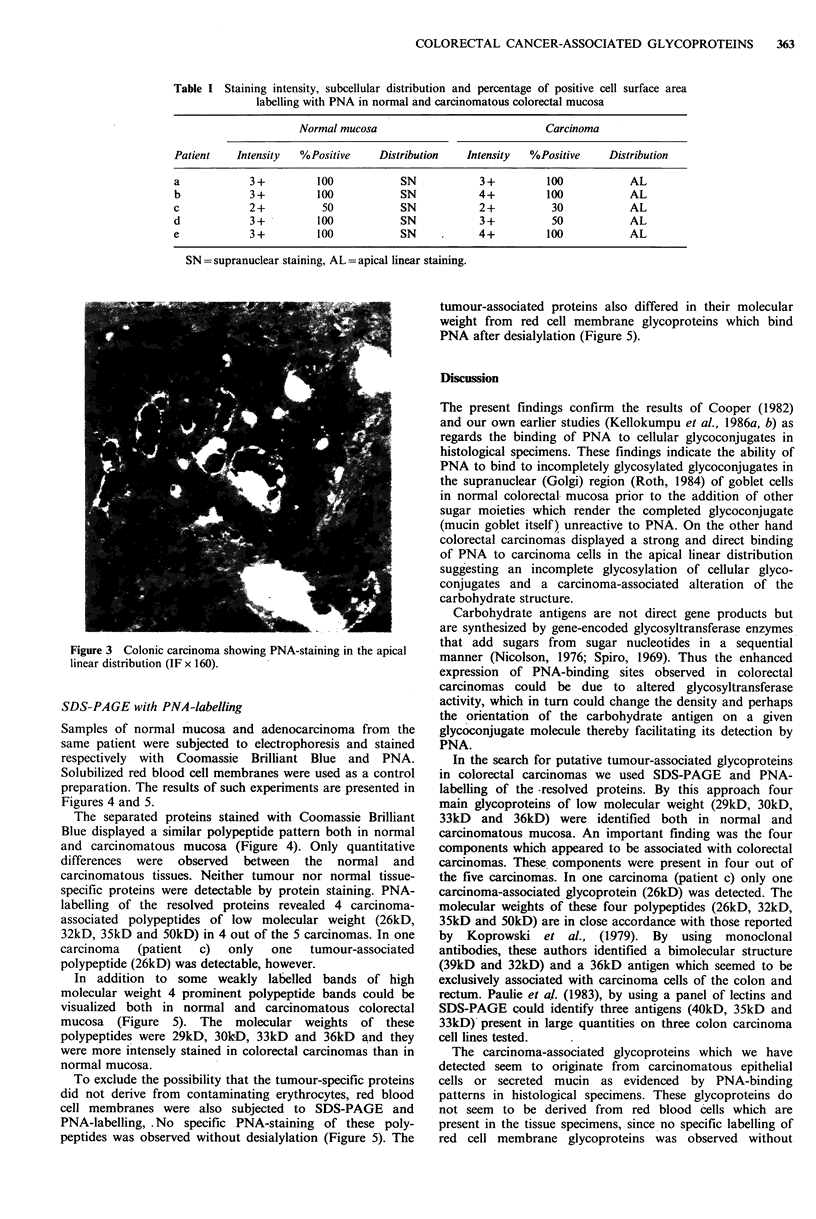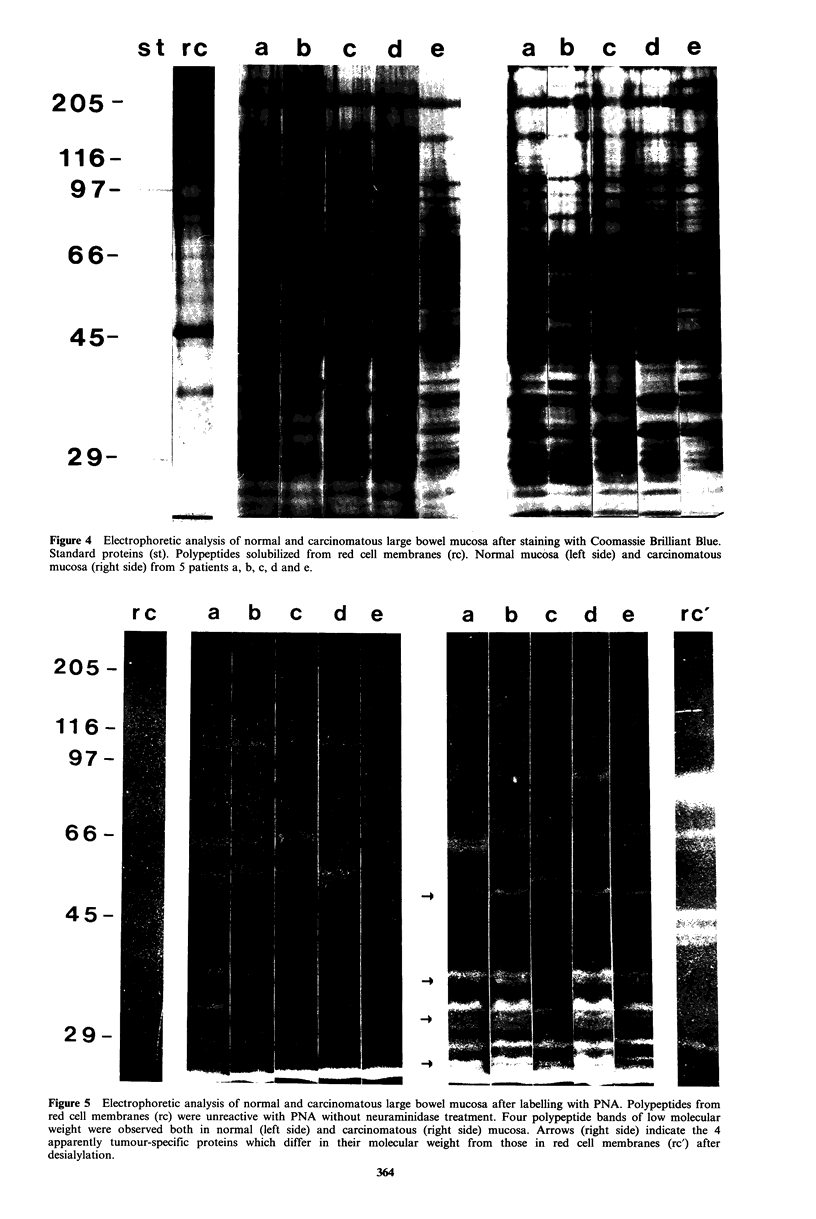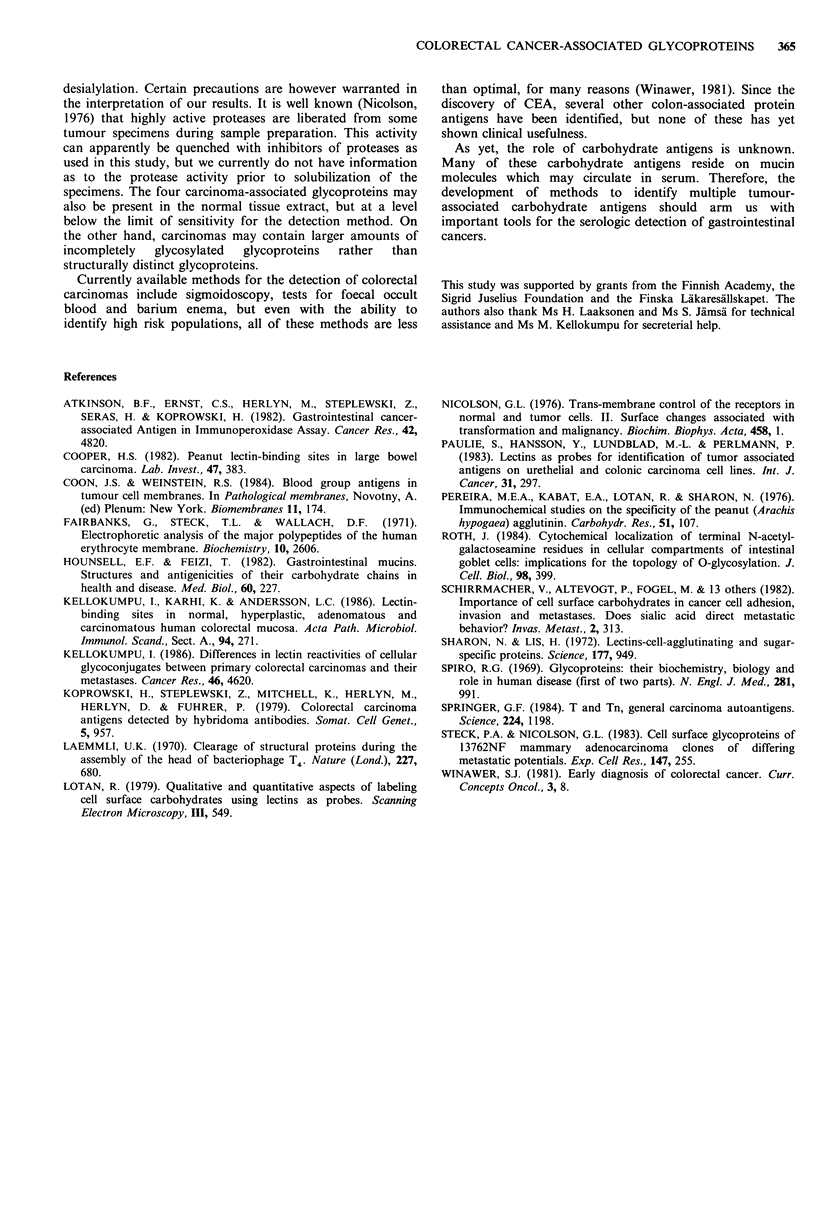# Identification of glycoproteins expressing tumour-associated PNA-binding sites in colorectal carcinomas by SDS-GEL electrophoresis and PNA-labelling.

**DOI:** 10.1038/bjc.1987.73

**Published:** 1987-04

**Authors:** I. Kellokumpu, S. Kellokumpu, L. C. Andersson

## Abstract

**Images:**


Br. J. Cancer (1987), 55, 361–365 © The Macmillan Press Ltd., 1987

### Identification of glycoproteins expressing tumour-associated PNA-binding sites in colorectal carcinomas by SDS-GEL electrophoresis and PNA-labelling

I. Kellokumpu[1], S. Kellokumpu[2] and L.C. Andersson[3]

[1]*Department of Surgery, Helsinki University Central Hospital;* [2]*Department of Anatomy, University of Oulu and* [3]*Department of Pathology, University of Helsinki, Finland.*

**Summary** Many tumour-specific antigens in gastrointestinal cancers have carbohydrate immunodeterminants. These epitopes can be identified by lectins and monoclonal antibodies. By using fluorescein-isothiocyanate (FITC)-conjugated peanut agglutinin (PNA) and sodium dodecyl sulfate-polyacrylamide gel electrophoresis (SDS-PAGE) we have investigated glycoproteins carrying altered carbohydrate epitopes in normal and carcinomatous human colorectal mucosa.

In normal mucosa PNA stained goblet cell glycoconjugates in the supranuclear (Golgi) distribution. After neuraminidase pretreatment PNA stained actual mucin goblet itself at all levels of the crypts. Colorectal carcinomas displayed a strong and direct binding of PNA to apical cell membranes of carcinomatous cells and intraluminal secretions.

Analysis of the glycoproteins by SDS-PAGE and PNA-labelling revealed four carcinoma-associated glycoproteins (26kD, 32kD, 35kD and 50kD). In addition, four glycoproteins (29kD, 30kD, 33kD and 36kD) common to normal and carcinomatous colorectal mucosa could be identified. All of these glycoproteins differed in their molecular weight from those in red cell controls which bind PNA only after desialylation.

The study shows that the expression of PNA-binding sites in colorectal carcinomas signifies a cancer-associated carbohydrate alteration. Four carcinoma-associated glycoprotein antigens could be detected by this lectin. The antigens we have identified might be useful in the isolation and purification of more selective reagents for the serologic detection of colorectal cancer.

The search for tumour-specific markers of gastrointestinal cancer has revealed that many of the promising new markers have carbohydrate immunodeterminants (ABO antigens, Lewis[a] and Lewis[b] antigens, T-antigen and CA 19-9) (Hounsell *et al.*, 1982; Coon & Weinstein, 1984; Springer, 1984; Atkinson *et al.*, 1982).

The carbohydrate binding site of peanut agglutinin (PNA) is most complementary to the sequence galactose $\beta$1-3-N-actylgalactosamine (Pereira *et al.*, 1976), the immunodeterminant structure of T-antigen, which is commonly expressed in many human carcinomas but not in normal tissue where it is covered by sialic acid residues (Coon & Weinstein, 1984; Springer, 1984). According to Pereira *et al.*, (1976) PNA also demonstrates specificity to a lesser degree for other terminal, nonreducing galactosyl-containing oligosaccharides, particularly for the structure galactose $\beta$1-4-N-acetylglucosamine, galactose $\beta$1-3-N-acetylglucosamine and galactose.

The molecular functions of T-antigen at a cellular level are largely unknown but its presence in human carcinomas frequently correlates with the carcinomas' aggressiveness (Springer, 1984). In addition, cell surface glycoconjugates from a highly metastatic rat mammary adenocarcinoma (Steck & Nicolson, 1983) and from murine Eb lymphoma cells (Schirrmacher *et al.*, 1982) are known to strongly express PNA-binding sites.

Sodium dodecyl sulfate-polyacrylamide gel electrophoresis (SDS-PAGE) is being widely used for detecting glycoproteins (Laemmli, 1970). Several groups have compared normal and transformed cells in culture, but little work has been done directly with solid tumours.

We therefore have compared normal and carcinomatous human colorectal mucosa with SDS-PAGE and PNA-labelling in order to detect possible tumour-associated glycoproteins. Here we report the identification of four carcinoma-associated glycoproteins with specific PNA-binding sites.

Correspondence: I. Kellokumpu.
Received 25 July 1986; and in revised form 29 October 1986.

### Materials and methods

### *Tissue samples*

Fresh specimens of normal and carcinomatous colorectal mucosa, obtained at surgery from 5 patients with colorectal carcinoma were processed without delay. Half of each specimen of normal colorectal mucosa or carcinoma was immediately snapfrozen in liquid nitrogen and stored at −70°C until use. The other half of each specimen was fixed in formalin and embedded in paraffin for conventional histological staining procedures and lectin histochemistry.

The histopathological diagnosis of colorectal carcinomas was confirmed and the histological grade of the tumours was assessed using haematoxylin-eosin stained tissue sections. The samples of normal colorectal mucosa consisted of resection surfaces, at a distance of at least 10 cm from the adjacent carcinoma. The 5 colorectal carcinomas included one rectal carcinoma, 2 sigmoid carcinomas, one carcinoma from the ascending colon and one from the caecum. Two tumours were well differentiated and 3 tumours were moderately-well differentiated.

### *PNA-staining procedures in histological specimens and fluorescent microscopy*

Fluorescein-isothiocyanate (FITC)-conjugated peanut agglutinin (PNA) from *Arachis hypogaea*, specific for galactose $\beta$1-3-N-acetylgalactosamine, was obtained from Vector laboratories, Burlingame, CA (Sharon *et al.*, 1972; Lotan, 1979). FITC-lectin solution was prepared in PBS at a concentration of 1 $\mu$mg ml$^{-1}$. The working concentration used in this study was 200 $\mu$g ml$^{-1}$ in PBS. The sugar binding specificity of PNA was confirmed by the absence of fluorescent staining in tissue sections after preincubation of the lectin with a specific 0.2 M galactose solution, and by the positive fluorescent staining subsequent to preincubation with non-specific 0.2 M solution of $\alpha$-methylmannoside.

Five $\mu$m thick tissue sections were cut from paraffin-embedded tissue specimens. These were then mounted on glass slides, deparaffinized by two 10 min washes in xylene and rehydrated by serial 2 min washes in graded alcohols, followed by three 5 min washes in PBS (pH 7.2). The hydrated tissue sections were covered by FITC-PNA in a moist chamber, at room temperature for 20 min, the unbound lectin was then washed off with PBS and the slides were mounted with PBS-glycerol (pH 8.0). Lectin binding was also studied after desialylation of the specimens. Tissue sections were covered by 50 $\mu$l of Vibrio cholerae neuraminidase 1 UI ml$^{-1}$ (Test-Neuraminidase®, Behringwerke AG, West Germany) in a moist chamber, at room temperature for 30 min and then washed in PBS before being labelled with PNA. The stained slides were examined with a Zeiss epifluorescence microscope. The entire tissue section was examined, and the overall percentage of positively stained tumour cells for the entire section was estimated. The intensity of labelling was scored from 0 (absent) to 4+ (strong fluorescence). Interstitial stroma and red blood cells served as intrinsic controls.

*Analysis by sodium dodecyl sulfate-polyacrylamide gel electrophoresis (SDS-PAGE) and PNA-labelling*

About 100 mg of normal and carcinoma tissues from each patient were homogenized with a glass homogenizer in 400 $\mu$l of 1% Triton X-100 (in 10 mM PBS, pH 7.4) containing a mixture of protease inhibitors (phenylmethylsulfonyl fluoride, phenanthroline and N-ethylmaleimide, 2 mM each) (Sigma Chemical Co., St. Louis, MO, USA). Aliquots of the homogenates were mixed with an equal volume of Laemmli-sample buffer and boiled for 10 min in the presence of 2% mercaptoethanol. Anfter centrifugation for 10 min at 10,000 *g* in an Eppendorff-centrifuge, the supernatants were taken and stored frozen (−20°C) until use.

Aliquots (50 $\mu$l) of each sample were analyzed using a discontinuous system of Laemmli (1970), which comprised of a 9% separating gel and a 5% stacking gel. The separated proteins and molecular weight standards (Sigma MW-SDS-200) were stained with Coomassie Brilliant Blue according to Fairbanks *et al.* (1971). The separated proteins were electro-eluted from the slab gels with a constant current of 100 mA for 6 h at +4°C, using Bio-Rad's transfer apparatus and 25 mM Tris-HCl/192 mM Glycin (pH 8.3) as a transfer medium.

Following electroelution, nitrocellulose sheets were rinsed with PBS and water, and then air dried. For lectin overlay, the sheets were incubated in PBS for 30 min before subjecting to 2% bovine serum albumin (in PBS) (Sigma Chemical Co., St. Louis, MO, USA) for 2 h at +20°C to block background labelling. After rinsing with PBS, the sheets were incubated with fluorescein-conjugated peanut agglutinin (Vector Laboratories, Burlingame, CA, USA) at 200 $\mu$g ml$^{-1}$ in PBS containing 0.01% $NaN_3$ (sodium azide) for 12–16 h at +4°C. Unbound lectin was removed by washing three times, 10 min each, with 0.1% Triton X-100/PBS and finally with PBS alone.

The labelled proteins were visualized by irradiation under UV-light (366 nm) and photographed using a 400 nm UV-filter and Kodak Tri-X Pan film. To exlude that the labelled proteins are derived from contaminating blood cells, red blood cell membranes were solubilized identically and subjected to lectin overlay either with or without desialylation in 50 mM $H_2SO_4$-solution for 1 h at +80°C.

### Results

*PNA-staining patterns in histological specimens*

The specimens of normal mucosa consisted of mucosa, muscularis mucosa and varying amounts of submucosal connective tissue. Within the mucosa goblet cell epithelial layer rests upon the basement membrane and underlying lamina propria. The samples of carcinomatous tissue exhibited various degree of mucin production and fibrous stromal reaction.

**Figure 1** Normal mucosa showing the PNA-fluorescence in the supra-nuclear (Golgi) region, with negative mucin goblets (IF × 250).

**Figure 2** Section of normal colonic mucosa showing PNA-binding localized to goblet cell mucin after neuraminidase treatment (IF × 160).

The results of PNA-staining in histological specimens are presented in Table I. In all specimens of normal mucosa PNA stained cellular glycoconjugates in the supranuclear (Golgi) region of goblet cells (Figure 1). After neuraminidase treatment the PNA-reactivity was found in goblet cell mucin (mucin goblet itself) at all levels of the crypt (Figure 2).

In colorectal carcinomas PNA-staining consisted of a fine linear fluorescence of apical cell membranes in carcinomatous glands and fluorescent intraluminal secretions (Figure 3). Intralesional heterogeneity, resulting in areas of fluorescence negative and fluorescence positive carcinomatous glands was observed in two carcinomas (patients c and d, Table I).

Inhibition studies with 0.2 M galactose solution confirmed the sugar binding specificity of PNA. Interstitial stroma showed minimal to no background staining. Red blood cells demonstrated a specific binding of PNA only after neuraminidase treatment of tissue sections.

**Table I** Staining intensity, subcellular distribution and percentage of positive cell surface area labelling with PNA in normal and carcinomatous colorectal mucosa

| | *Normal mucosa* | | | *Carcinoma* | | |
|---|---|---|---|---|---|---|
| *Patient* | *Intensity* | *%Positive* | *Distribution* | *Intensity* | *%Positive* | *Distribution* |
| a | 3+ | 100 | SN | 3+ | 100 | AL |
| b | 3+ | 100 | SN | 4+ | 100 | AL |
| c | 2+ | 50 | SN | 2+ | 30 | AL |
| d | 3+ | 100 | SN | 3+ | 50 | AL |
| e | 3+ | 100 | SN | 4+ | 100 | AL |

SN = supranuclear staining, AL = apical linear staining.

**Figure 3** Colonic carcinoma showing PNA-staining in the apical linear distribution (IF x 160).

### *SDS-PAGE with PNA-labelling*

Samples of normal mucosa and adenocarcinoma from the same patient were subjected to electrophoresis and stained respectively with Coomassie Brilliant Blue and PNA. Solubilized red blood cell membranes were used as a control preparation. The results of such experiments are presented in Figures 4 and 5.

The separated proteins stained with Coomassie Brilliant Blue displayed a similar polypeptide pattern both in normal and carcinomatous mucosa (Figure 4). Only quantitative differences were observed between the normal and carcinomatous tissues. Neither tumour nor normal tissue-specific proteins were detectable by protein staining. PNA-labelling of the resolved proteins revealed 4 carcinoma-associated polypeptides of low molecular weight (26kD, 32kD, 35kD and 50kD) in 4 out of the 5 carcinomas. In one carcinoma (patient c) only one tumour-associated polypeptide (26kD) was detectable, however.

In addition to some weakly labelled bands of high molecular weight 4 prominent polypeptide bands could be visualized both in normal and carcinomatous colorectal mucosa (Figure 5). The molecular weights of these polypeptides were 29kD, 30kD, 33kD and 36kD and they were more intensely stained in colorectal carcinomas than in normal mucosa.

To exclude the possibility that the tumour-specific proteins did not derive from contaminating erythrocytes, red blood cell membranes were also subjected to SDS-PAGE and PNA-labelling, . No specific PNA-staining of these polypeptides was observed without desialylation (Figure 5). The tumour-associated proteins also differed in their molecular weight from red cell membrane glycoproteins which bind PNA after desialylation (Figure 5).

### Discussion

The present findings confirm the results of Cooper (1982) and our own earlier studies (Kellokumpu *et al.*, 1986*a*, *b*) as regards the binding of PNA to cellular glycoconjugates in histological specimens. These findings indicate the ability of PNA to bind to incompletely glycosylated glycoconjugates in the supranuclear (Golgi) region (Roth, 1984) of goblet cells in normal colorectal mucosa prior to the addition of other sugar moieties which render the completed glycoconjugate (mucin goblet itself) unreactive to PNA. On the other hand colorectal carcinomas displayed a strong and direct binding of PNA to carcinoma cells in the apical linear distribution suggesting an incomplete glycosylation of cellular glycoconjugates and a carcinoma-associated alteration of the carbohydrate structure.

Carbohydrate antigens are not direct gene products but are synthesized by gene-encoded glycosyltransferase enzymes that add sugars from sugar nucleotides in a sequential manner (Nicolson, 1976; Spiro, 1969). Thus the enhanced expression of PNA-binding sites observed in colorectal carcinomas could be due to altered glycosyltransferase activity, which in turn could change the density and perhaps the orientation of the carbohydrate antigen on a given glycoconjugate molecule thereby facilitating its detection by PNA.

In the search for putative tumour-associated glycoproteins in colorectal carcinomas we used SDS-PAGE and PNA-labelling of the resolved proteins. By this approach four main glycoproteins of low molecular weight (29kD, 30kD, 33kD and 36kD) were identified both in normal and carcinomatous mucosa. An important finding was the four components which appeared to be associated with colorectal carcinomas. These components were present in four out of the five carcinomas. In one carcinoma (patient c) only one carcinoma-associated glycoprotein (26kD) was detected. The molecular weights of these four polypeptides (26kD, 32kD, 35kD and 50kD) are in close accordance with those reported by Koprowski *et al.*, (1979). By using monoclonal antibodies, these authors identified a bimolecular structure (39kD and 32kD) and a 36kD antigen which seemed to be exclusively associated with carcinoma cells of the colon and rectum. Paulie *et al.* (1983), by using a panel of lectins and SDS-PAGE could identify three antigens (40kD, 35kD and 33kD) present in large quantities on three colon carcinoma cell lines tested.

The carcinoma-associated glycoproteins which we have detected seem to originate from carcinomatous epithelial cells or secreted mucin as evidenced by PNA-binding patterns in histological specimens. These glycoproteins do not seem to be derived from red blood cells which are present in the tissue specimens, since no specific labelling of red cell membrane glycoproteins was observed without

**Figure 4** Electrophoretic analysis of normal and carcinomatous large bowel mucosa after staining with Coomassie Brilliant Blue. Standard proteins (st). Polypeptides solubilized from red cell membranes (rc). Normal mucosa (left side) and carcinomatous mucosa (right side) from 5 patients a, b, c, d and e.

**Figure 5** Electrophoretic analysis of normal and carcinomatous large bowel mucosa after labelling with PNA. Polypeptides from red cell membranes (rc) were unreactive with PNA without neuraminidase treatment. Four polypeptide bands of low molecular weight were observed both in normal (left side) and carcinomatous (right side) mucosa. Arrows (right side) indicate the 4 apparently tumour-specific proteins which differ in their molecular weight from those in red cell membranes (rc′) after desialylation.

desialylation. Certain precautions are however warranted in the interpretation of our results. It is well known (Nicolson, 1976) that highly active proteases are liberated from some tumour specimens during sample preparation. This activity can apparently be quenched with inhibitors of proteases as used in this study, but we currently do not have information as to the protease activity prior to solubilization of the specimens. The four carcinoma-associated glycoproteins may also be present in the normal tissue extract, but at a level below the limit of sensitivity for the detection method. On the other hand, carcinomas may contain larger amounts of incompletely glycosylated glycoproteins rather than structurally distinct glycoproteins.

Currently available methods for the detection of colorectal carcinomas include sigmoidoscopy, tests for foecal occult blood and barium enema, but even with the ability to identify high risk populations, all of these methods are less than optimal, for many reasons (Winawer, 1981). Since the discovery of CEA, several other colon-associated protein antigens have been identified, but none of these has yet shown clinical usefulness.

As yet, the role of carbohydrate antigens is unknown. Many of these carbohydrate antigens reside on mucin molecules which may circulate in serum. Therefore, the development of methods to identify multiple tumour-associated carbohydrate antigens should arm us with important tools for the serologic detection of gastrointestinal cancers.

This study was supported by grants from the Finnish Academy, the Sigrid Juselius Foundation and the Finska Läkaresällskapet. The authors also thank Ms H. Laaksonen and Ms S. Jämsä for technical assistance and Ms M. Kellokumpu for secreterial help.

**References**

ATKINSON, B.F., ERNST, C.S., HERLYN, M., STEPLEWSKI, Z., SERAS, H. & KOPROWSKI, H. (1982). Gastrointestinal cancer-associated Antigen in Immunoperoxidase Assay. *Cancer Res.*, **42,** 4820.

COOPER, H.S. (1982). Peanut lectin-binding sites in large bowel carcinoma. *Lab. Invest.*, **47,** 383.

COON, J.S. & WEINSTEIN, R.S. (1984). Blood group antigens in tumour cell membranes. In *Pathological membranes*, Novotny, A. (ed) Plenum: New York. *Biomembranes* **11,** 174.

FAIRBANKS, G., STECK, T.L. & WALLACH, D.F. (1971). Electrophoretic analysis of the major polypeptides of the human erythrocyte membrane. *Biochemistry*, **10,** 2606.

HOUNSELL, E.F. & FEIZI, T. (1982). Gastrointestinal mucins. Structures and antigenicities of their carbohydrate chains in health and disease. *Med. Biol.*, **60,** 227.

KELLOKUMPU, I., KARHI, K. & ANDERSSON, L.C. (1986). Lectin-binding sites in normal, hyperplastic, adenomatous and carcinomatous human colorectal mucosa. *Acta Path. Microbiol. Immunol. Scand.*, Sect. A., **94,** 271.

KELLOKUMPU, I. (1986). Differences in lectin reactivities of cellular glycoconjugates between primary colorectal carcinomas and their metastases. *Cancer Res.*, **46,** 4620.

KOPROWSKI, H., STEPLEWSKI, Z., MITCHELL, K., HERLYN, M., HERLYN, D. & FUHRER, P. (1979). Colorectal carcinoma antigens detected by hybridoma antibodies. *Somat. Cell Genet.*, **5,** 957.

LAEMMLI, U.K. (1970). Clearage of structural proteins during the assembly of the head of bacteriophage $T_4$. *Nature* (*Lond.*), **227,** 680.

LOTAN, R. (1979). Qualitative and quantitative aspects of labeling cell surface carbohydrates using lectins as probes. *Scanning Electron Microscopy*, **III,** 549.

NICOLSON, G.L. (1976). Trans-membrane control of the receptors in normal and tumor cells. II. Surface changes associated with transformation and malignancy. *Biochim. Biophys. Acta*, **458,** 1.

PAULIE, S., HANSSON, Y., LUNDBLAD, M.-L. & PERLMANN, P. (1983). Lectins as probes for identification of tumor associated antigens on urethelial and colonic carcinoma cell lines. *Int. J. Cancer*, **31,** 297.

PEREIRA, M.E.A., KABAT, E.A., LOTAN, R. & SHARON, N. (1976). Immunochemical studies on the specificity of the peanut (*Arachis hypogaea*) agglutinin. *Carbohydr. Res.*, **51,** 107.

ROTH, J. (1984). Cytochemical localization of terminal N-acetyl-galactoseamine residues in cellular compartments of intestinal goblet cells: implications for the topology of O-glycosylation. *J. Cell. Biol.*, **98,** 399.

SCHIRRMACHER, V., ALTEVOGT, P., FOGEL, M. & 13 others (1982). Importance of cell surface carbohydrates in cancer cell adhesion, invasion and metastases. Does sialic acid direct metastatic behavior? *Invas. Metast.*, **2,** 313.

SHARON, N. & LIS, H. (1972). Lectins-cell-agglutinating and sugar-specific proteins. *Science*, **177,** 949.

SPIRO, R.G. (1969). Glycoproteins: their biochemistry, biology and role in human disease (first of two parts). *N. Engl. J. Med.*, **281,** 991.

SPRINGER, G.F. (1984). T and Tn, general carcinoma autoantigens. *Science*, **224,** 1198.

STECK, P.A. & NICOLSON, G.L. (1983). Cell surface glycoproteins of 13762NF mammary adenocarcinoma clones of differing metastatic potentials. *Exp. Cell Res.*, **147,** 255.

WINAWER, S.J. (1981). Early diagnosis of colorectal cancer. *Curr. Concepts Oncol.*, **3,** 8.